# Rhodamine-Anchored Polyacrylamide Hydrogel for Fluorescent Naked-Eye Sensing of Fe^3+^

**DOI:** 10.3390/molecules28186572

**Published:** 2023-09-11

**Authors:** Dandan Jiang, Minghao Zheng, Xiaofan Ma, Yingzhen Zhang, Shaohua Jiang, Juanhua Li, Chunmei Zhang, Kunming Liu, Liqing Li

**Affiliations:** 1 Jiangxi Provincial Key Laboratory of Functional Molecular Materials Chemistry, Faculty of Materials Metallurgy and Chemistry, Jiangxi University of Science and Technology, Ganzhou 341000, China; 18720211955@163.com (D.J.); 18870773639@163.com (M.Z.); m15779018433@163.com (Y.Z.); lijuanhua@jxust.edu.cn (J.L.); 2 Jiangsu Co-Innovation Center of Efficient Processing and Utilization of Forest Resources, International Innovation Center for Forest Chemicals and Materials, College of Materials Science and Engineering, Nanjing Forestry University, Nanjing 210037, China; xiaofanma@njfu.edu.cn (X.M.); shaohua.jiang@njfu.edu.cn (S.J.); 3 Institute of Materials Science and Devices, School of Materials Science and Engineering, Suzhou University of Science and Technology, Suzhou 215009, China; cmzhang@usts.edu.cn

**Keywords:** hydrogel sensor, rhodamine, selectivity, naked-eye detection, free radical polymerization

## Abstract

A fluorescent and colorimetric poly (acrylamide)-based copolymer probe **P(AAm-co-RBNCH)** has been designed via free radical polymerization of a commercial acrylamide monomer with a rhodamine-functionalized monomer **RBNCH**. Metal ion selectivity of **RBNCH** was investigated by fluorescence and colorimetric spectrophotometry. Upon addition of Fe^3+^, a visual color change from colorless to red and a large fluorescence enhancement were observed for the ring-opening of the rhodamine spirolactam mechanism. The monomer gives a sensitive method for quantitatively detecting Fe^3+^ in the linear range of 100–200 μM, with a limit of detection as low as 27 nM and exhibiting high selectivity for Fe^3+^ over 12 other metal ions. The hydrogel sensor was characterized by FTIR, and the effects of **RBNCH** amount on gel content and swelling properties were explored. According to the recipe of 1.0 mol% **RBNCH** to the total monomers, the fabricated hydrogel sensor displayed a good swelling property and reversibility performance and has potential for application in the imaging of Fe^3+^ level in industrial wastewater.

## 1. Introduction

Owing to their three-dimensional hydrophilic polymeric networks, hydrogels can easily swell, up to hundreds or thousands of times their original size, in aqueous media and respond smartly to electricity, light, pH or temperature variations [1,2,3,4,5,6]. Multiple hydrogels have played extremely important roles in the fields of antimicrobial materials, drug delivery systems, tissue engineering and superabsorbents [7,8,9,10,11]. Additionally, their transparent and manipulative properties make these hydrogels excellent candidates for smart sensing materials with colorimetric or fluorescent signal change [12,13].

Chemosensors possess simpler operation, higher selectivity and more rapid response compared with instrumental assays such as atomic absorption spectrometry (AAS), inductively coupled plasma-atomic emission spectroscopy (ICP-AES), ion selective electrodes (ISE) and UV-vis spectrophotometry [14,15,16]. Nowadays, the construction of chemosensors for the specific heavy metal ions that are essential in living systems or have a toxic impact on our environment attracts considerable interest. Fe^3+^, as a vital physiologically relevant metal ion, is indispensable in the process of cellular metabolism, oxygen metabolism, enzyme catalysis and protein synthesis [17,18,19]. An excess or deficiency of Fe^3+^ in a living organism can lead to physiological diseases including anemia, heart failure, hepatitis and neurodegenerative disease [20,21,22]. In the last decade, colorimetric and fluorescent probes based on coumarin, BODIPY, dansyl chloride, and pyrene have been developed for the quantitative determination of Fe^3+^ [23,24,25,26]. Among diverse organic fluorophores, rhodamines are noted for their high fluorescence quantum yield, large absorption coefficient and wide wavelength range. In recent years rhodamine-based sensors have been widely applied to recognize metal ions [27,28,29,30,31,32,33,34]. However, most of these sensors are insoluble in water due to their hydrophobicity. Although several hydrophilic molecular sensors have been reported, their reusability is poor due to inconvenience [35,36,37]. Crosslinked hydrogels encompass hydrophilic and insoluble properties, allowing researchers to gain insight into the design of reusable and visible sensors of specific heavy metal ions. In 2012, Lin et al., synthesized thiourea functionalized polyacrylamide hydrogel sensors containing polystyrene photonic crystals for the detection of Cd^2+^ [38]. Subsequently, a 5, 6-dicarboxylic fluorescein cross-linked amine-functionalized polyacrylamide hydrogel as visual volumetric sensor for Cu^2+^ recognition was designed by Wu’s group [39]. Recently, Qu and coworkers reported a rhodamine-immobilized optical hydrogel for the selective sensing of Hg^2+^ [40]. In addition, DNA cross-linked hydrogels were synthesized based on the acrydite-DNA strands, and these act as capillary sensors for Pb^2+^ monitoring in the literature [41]. Apart from the abovementioned sensitive sensors for several metal ions, developing a simple-to-prepare and reusable hydrogel sensor for the detection of Fe^3+^ would greatly enrich the types of smart sensing materials. In 2013, Ozay et al., developed a colorimetric hydrogel sensor for Fe^3+^ recognition based on the Rhodamine G fluorophore [42]. Liu et al., reported a portable Fe^3+^ hydrogel sensor based on the urea-linked functionalized rhodamine monomers [43].

In this study, a transparent hydrogel sensor that shows fluorescence in response to selective sensing Fe^3+^ was synthesized and characterized. The rhodamine fluorophore was anchored to the side chain of polyacrylamide by radical co-polymerization using the synthesized novel monomer **RBNCH**. When exposed to Fe^3+^, the color of the hydrogel turns to shiny red under the UV light at 365 nm, and the color intensity is associated with the Fe^3+^ ion concentration. In addition, we investigated the reusability of the hydrogel sensor using ethylenediamine (EDA) solution.

## 2. Results and Discussion

### 2.1. Design and Synthesis

**Figure 1:**
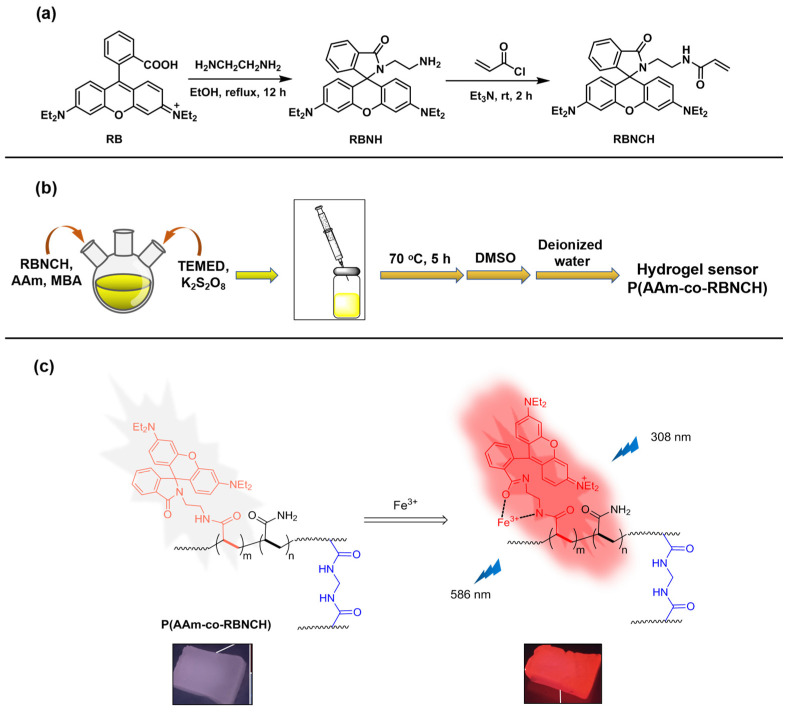
Design and synthesis of the hydrogel sensors. (**a**) Synthesis route for the monomer **RBNCH**; (**b**) preparation route for the hydrogel sensor; (**c**) Fe^3+^ recognition mechanism of the hydrogel sensor.

In the strategy for design of fluorescence sensors, rational selection of organic dyes as the signaling subunit is very important. Rhodamine skeleton has been widely utilized as the fluorophore based on the fluorescence produced by the structure change from spirocyclic ring to open ring form. The designed monomer **RBNCH** is nonfluorescent and colorless in the spirolactam structure. In the case of the **RBNCH** complex with Fe^3+^, the lactam ring of the spirane structure opens up and leads to strong fluorescence emission. Conceivably, the rhodamine structure anchored to the copolymer main chain could also enable the hydrogel **P(AAm-co-RBNCH)** to allow selective recognition of Fe^3+^ (Figure 1). The monomer **RBNCH** and intermediate **RBNH** were synthesized from the commercially available and inexpensive rhodamine B as shown in Figure 1; structures of both compounds were confirmed by NMR and HRMS (Figures S1–S3, Supplementary Materials).

### 2.2. Fluorescent Spectra Studies of **RBNCH**

**Figure 2:**
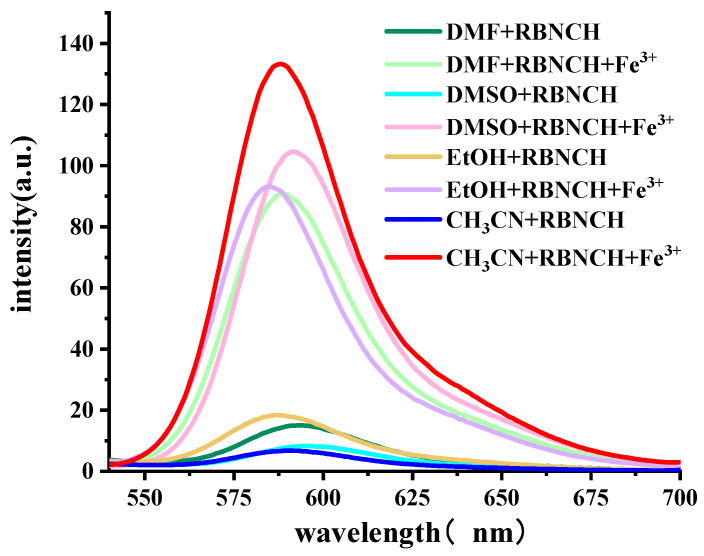
Fluorescence emission spectra (λ_ex_ = 308 nm) of the sensing system containing 50 μM **RBNCH**, with or without 100 μM Fe^3+^ in indicated solvents.

To verify this design hypothesis, we tested the fluorescence response of **RBNCH** to Fe^3+^. The fluorescence spectra of the probe for Fe^3+^ detection in various solvents is shown in Figure 2. The solutions of **RBNCH** alone exhibited weak fluorescence emission above 500 nm, while the maximum fluorescence enhancement could be observed in CH_3_CN upon addition of Fe^3+^, which indicates the relatively significant solvent effect in the sensing system.

Considering the poor solubility of the monomer in water, we optimized the CH_3_CN/H_2_O ratio of the detection system. At room temperature (25 °C), it can be found that the fluorescence intensity decreased when elevating H_2_O content in the solvent, achieving the highest enhancement with CH_3_CN/H_2_O = 9:1, *v*/*v* (Figure S4, Supplementary Materials). The alteration of fluorescence against various incubation times was also examined (Figure S5, Supplementary Materials). After adding Fe^3+^, the fluorescence intensity of the sensing system increased gradually with an extended incubation time, reaching a steady state at 1.5 h. Hence, we chose 1.5 h as the best incubation time for the subsequent experiments. In this scenario, the subsequent analysis was carried out in testing solutions (CH_3_CN/H_2_O = 9:1, *v*/*v*) with 1.5 h as incubation time. It is worth noting that the color change begins after 5 min, and the fluorescent intensity increased to 200 a.u. within 20 min (the moment at which color change is detected by eye). Therefore, it can be considered that the probe can produce a significant fluorescence response within 20 min, which gives it a rapid response speed.

**Figure 3:**
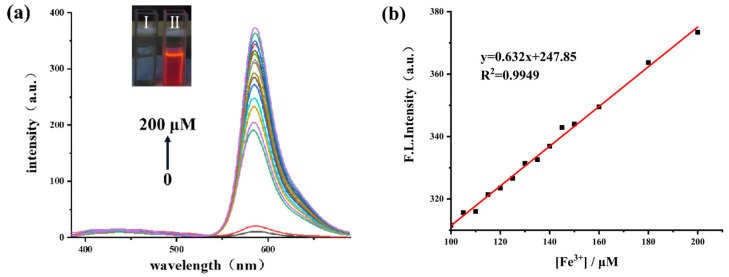
(**a**) The fluorescence spectra of **RBNCH** (50 μM) before and after addition of Fe^3+^ (0–200 μM). Inset: photographs of 1 mM of **RBNCH** without Fe^3+^ (**I**) and with 1 mM of Fe^3+^ (**II**) under 365 nm UV illumination; (**b**) the linear correlations between fluorescence intensity at 586 nm andconcentration of Fe^3+^ (100–200 μM), λ_ex_ = 308 nm.

To test sensitivity, the presentation of fluorescent spectra with varied concentrations of Fe^3+^ was studied. After adding Fe^3+^ to the **RBNCH** solution, a dramatic increase was observed in the fluorescence intensity of the solution and the fluorescent emission maximum is centered at about 586 nm (Figure 3a). It was determined that the fluorescence intensity was proportional to the concentration of Fe^3+^ and a linear equation of *F_586nm_* = 247.85 + 0.632 [Fe^3+^] (R^2^ = 0.9949) was obtained, with a limit of detection (LOD) calculated to be as low as 27 nM, according to LOD = 3σ/*k* (where σ is the standard deviation of blank measurement and *k* is the slope between the fluorescence intensity versus Fe^3+^ concentration) (Figure 3b). The results above demonstrated that **RBNCH** is highly sensitive to Fe^3+^ and could be used to synthesize fluorescent sensing materials. Additionally, the synthesized monomer possesses higher sensitivity compared to the reported probes for monitoring Fe^3+^ (Table S1, Supplementary Materials).

**Figure 4:**
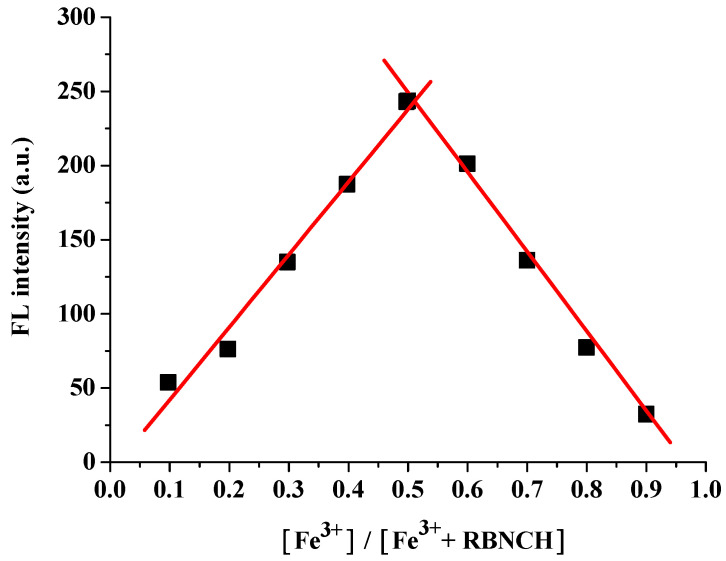
Job’s plot titration of **RBNCH** with Fe^3+^.

To further investigate the binding mode between **RBNCH** and Fe^3+^, a test solution with a total concentration of 100 μM and a ratio of [Fe^3+^]/([Fe^3+^] + [**RBNCH**]) ranging from 0.1 to 0.9 was prepared for fluorescence spectroscopy. The resulting Job’s plot is shown in Figure 4. It can be seen that the fluorescence intensity is maximum when the ratio is 0.5, which indicates that the binding value of the **RBNCH**-Fe^3+^ complex was 1:1.

**Figure 5:**
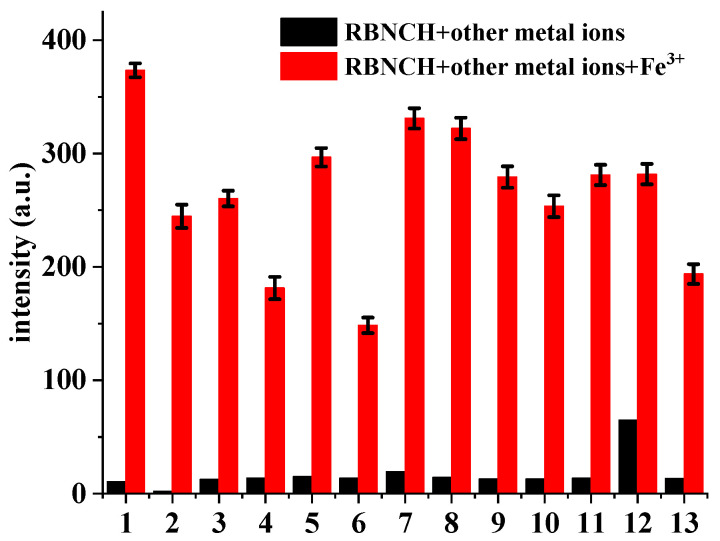
Fluorescence intensity changes of **RBNCH** (50 μM) upon addition of common metal ions: 1, none; 2, K^+^ (2 mM); 3, Na^+^ (2 mM); 4, Ca^2+^ (1 mM); 5, Mg^2+^ (1 mM); 6, Ni^2+^ (100 μM); 7, Cu^2+^ (100 μM); 8, Mn^2+^ (100 μM); 9, Pb^2+^ (100 μM); 10, Zn^2+^ (100 μM); 11, Pr^3+^ (100 μM); 12, Al^3+^ (100 μM); 13, Cd^2+^ (100 μM). Black bars: probe treated with the marked metal ions in the absence of Fe^3+^; red bars: probe treated with the marked metal ions followed by Fe^3+^ (100 μM). Each group of experiments was measured three times in parallel and error bars were determined based on their standard deviations.

The implementation of competitive experiments indicated that **RBNCH** gratifyingly enabled anti-interference capability against other metal ions (Figure 5). Only Fe^3+^ resulted in a remarkable fluorescence enhancement at 586 nm, whereas a range of common metal ions, including alkali and alkali-earth metal ions (K^+^, Na^+^, Ca^2+^ and Mg^2+^), transition metal ions (Zn^2+^, Ni^2+^, Cu^2+^, Mn^2+^, Pb^2+^, Cd^2+^ and Al^3+^) and rare-earth metal ions (Pr^3+^) merely caused a slight enhancement in the fluorescence, even when K^+^, Na^+^, Mg^2+^ and Ca^2+^ were added at millimolar levels.

### 2.3. Colorimetric Spectra Studies of **RBNCH**

**Figure 6:**
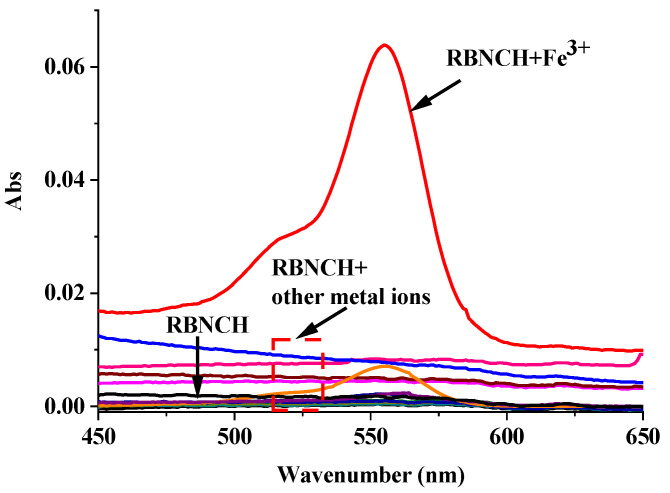
UV-vis spectra of **RBNCH** (50 μM) upon addition of common metal ions: K^+^ (2 mM), Na^+^ (2 mM), Ca^2+^ (1 mM), Mg^2+^ (1 mM), Ni^2+^ (100 μM), Cu^2+^ (100 μM), Mn^2+^ (100 μM), Pb^2+^ (100 μM), Zn^2+^ (100 μM), Fe^3+^ (100 μM), Pr^3+^ (100 μM), Al^3+^ (100 μM) and Cd^2+^ (100 μM).

The colorimetric response of **RBNCH** to metal ions is an important indicator of the naked-eye detection ability of the probe. A series of common metal ions (K^+^, Na^+^, Ca^2+^, Mg^2+^, Ni^2+^, Cu^2+^, Mn^2+^, Pb^2+^, Zn^2+^, Fe^3+^, Pr^3+^, Al^3+^ and Cd^2+^) were chosen to investigate the colorimetric selectivity of the **RBNCH**. As shown in Figure 6, in the absence of metal ions, the UV-vis spectra of **RBNCH** exhibited weak absorption. Upon the addition of Fe^3+^, a notable increase in absorbance at 555 nm is observed. In contrast, the addition of other metal ions does not result in significant spectral alterations or color changes under similar conditions. Consequently, the monomer **RBNCH** exhibits excellent colorimetric selectivity for Fe^3+^ and has the potential to be used as a naked-eye detection sensor.

**Figure 7:**
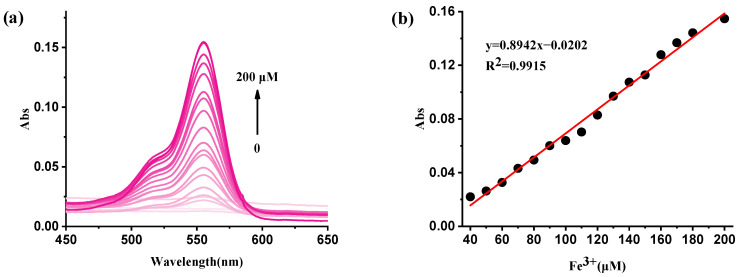
(**a**) The UV-vis spectra of **RBNCH** (50 μM) before and after addition of Fe^3+^ (0–200 μM); (**b**) the linear correlations between absorbance intensity at 555 nm and the concentration of Fe^3+^ (40–200 μM).

The UV/vis titration absorption spectra of **RBNCH** are presented in Figure 7a. The monomer **RBNCH** exhibits a weak visible-range absorption in the absence of Fe^3+^, which can be attributed to the fact that **RBNCH** exists as a spirocyclic structure in solution. Upon addition of Fe^3+^ to **RBNCH**, a significant shift occurs in the visible range. A distinct absorption band centered at 555 nm was observed when 0–200 μM Fe^3+^ was added. As the Fe^3+^ concentration increased from 40 to 200 μM, the absorption peak intensity at 555 nm gradually increased, exhibiting a linear relationship between A_555_ nm and [Fe^3+^] (R^2^ = 0.9915) (Figure 7b).

### 2.4. Characterization and Application of Rhodamine-Anchored Hydrogel Sensors

The utilization of molecular sensors in metal ion contaminant detection can be confronted with problems such as the terrible compatibility of water insoluble sensors, or ineffective reusability of hydrophilic sensors. Alternatively, hydrogels can swell in water and are renewable, making them promising for use as smart sensors in environmental monitoring. For the aforementioned reasons, **P(AAm-co-RBNCH)** hydrogels were synthesized, via free radical polymerization, using the different molar ratios of acrylamide (**AAm**) and **RBNCH** given in Table 1.

**Figure 8:**
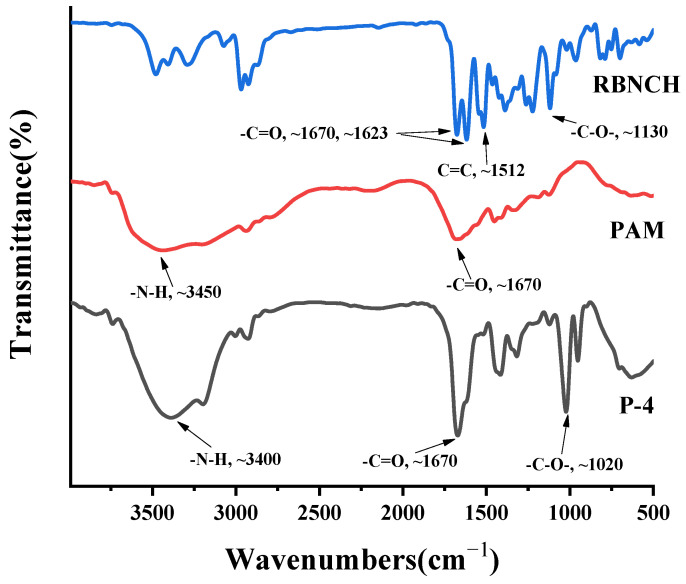
The FT-IR spectra of **RBNCH**, **PAM** and **P-4**.

To verify the success of co-polymerization, monomer **RBNCH**, homo-polymer Poly(acrylamide) (**PAM)** and hydrogel **P-4** were characterized by FT-IR spectra (Figure 8). In the IR spectra of **RBNCH**, the peaks at 1670 cm^−1^ and 1623 cm^−1^ represent the C=O stretching band for acryl amide and lactam group, respectively. The characteristic C=C and C-O stretching frequencies were observed at 1512 cm^−1^ and 1130 cm^−1^. In the spectrum of hydrogel **P-4**, the C=O band for the lactam group was covered by the stretching of acryl amide due to the low level of **RBNCH** in the co-polymer. However, the C-O stretching vibration at 1020 cm^−1^ can be clearly observed, while the characteristic C=C frequency at 1512 cm^−1^ disappears. Accordingly, the FT-IR spectra revealed that the **RBNCH** group was successfully polymerized into a hydrogel sensor.

**Figure 9:**
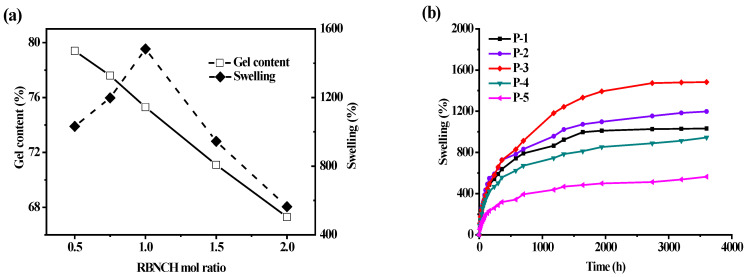
Studies on the water absorption capabilities of hydrogel sensors. (**a**) Effect of **RBNCH** mol ratio on gelation and swelling ratios; (**b**) hydrogels versus time in deionized water.

Gelation and swelling ratios were found to be directly related to the **RBNCH** ratio. As displayed in Figure 9a, the gelation ratio was inversely proportional to the added **RBNCH** in the co-polymer. When the content of **RBNCH** increased to 2 mol% (**P-5**), the gelation ratio fell to 67.3 ± 3.7%. From the perspective of rigidity and flexibility of molecular structure, the rhodamine-based monomer **RBNCH** possesses a rigid, highly conjugated xanthene structure as well as a considerable molecular weight, making copolymerization difficult. Therefore, the decreased gelation ratio may be due to the fact that cross-linking of two polymer chains was partially inhibited due to the rigid and sterically hindered rhodamine segment. With an increasing amount of **RBNCH**, the maximum swelling ratio of hydrogels in deionized water gradually increased and saturated when 1 mol% **RBNCH** was added. The reason for the lower swelling ability when the sensor monomer content exceeded 1 mol%, may be on account of incomplete polymerization and restricted cross-linking. Encompassing a compromise between adequate gelation and swelling properties, **P-3** was selected as the hydrogel sensor for the following tests. Swelling profiles of the hydrogels bearing different **RBNCH** content are shown in Figure 9b, which demonstrates that **P-3** has the best swelling rate and equilibrium swelling degree. The excellent water absorption capability of **P-3** may be due to the hydrophilic groups, such as -NH-, -CONH-, in the rhodamine skeleton, which could bind molecular water and expand the three-dimensional networks. Although previous reports on hydrogels have shown that an increase in rhodamine content leads to an increase in the swelling rate and equilibrium swelling degree. The lower swelling ratio of **P-4** and **P-5** in our work may derive from the limited three-dimensional network caused by the decreased gel content.

**Figure 10:**
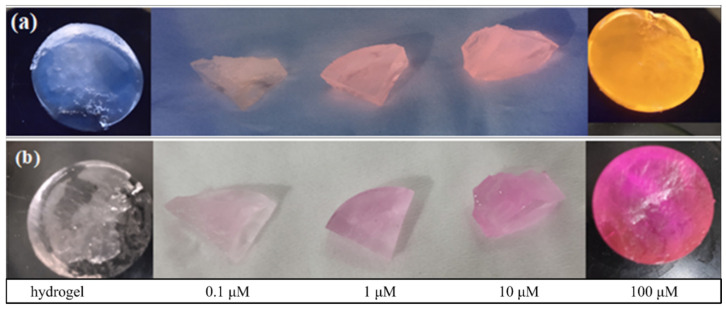
Naked-eye sensing of different concentrations of Fe^3+^ by rhodamine-anchored hydrogel sensor. (**a**) Under ultraviolet light at 365 nm; (**b**) under visible light.

The fluorescent response behavior of hydrogel sensors is displayed in Figure 10. It can be seen that the anchored rhodamine skeleton endows the copolymer with the ability of a colorimetric and fluorescence response to Fe^3+^. The detection limits of hydrogel sensors were determined in Fe^3+^ solutions ranging from 0.1 μM to 100 μM. The colorimetric and fluorescent intensities of hydrogels were enhanced with the increasing Fe^3+^ concentration, and the color change of the sensor can still be observed clearly, even in 0.1 μM Fe^3+^ solutions. The response time was related to the concentration of Fe^3+^. The color changes of a 3 mm thickness hydrogel in the presence of Fe^3+^ at 1 μM, 10 μM and 100 μM after 2.5 h, respectively.

**Figure 11:**
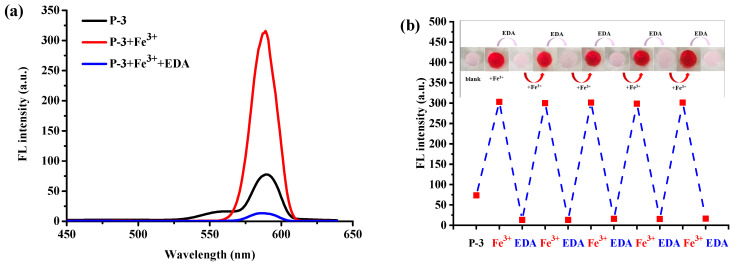
(**a**) Reversible changes in the fluorescence intensity of hydrogel sensor **P-3** after alternately adding Fe^3+^ (200 μM) and EDA (200 μM). (**b**) Repeatability of Fe^3+^-sensing behavior of the hydrogel sensor **P-3**. Inset: the color changes of **P-3** during the reuse process under ultraviolet light at 365 nm.

The reversibility performance was tested by putting a hydrogel sensor that has been sensing for Fe^3+^ into 200 μM ethylenediamine (**EDA**) solution. It can be seen in Figure 11a that an obvious fluorescent peak appears at 586 nm upon the addition of Fe^3+^ to the hydrogel **P-3**, and the fluorescence emission almost quenches after adding EDA to the detection solution. The fluorescent signal change derives from the stronger binding ability of EDA with Fe^3+^, which can capture Fe^3+^ from the **P(AAm-co-RBNCH)**-Fe^3+^ complex and lead to the rhodamine moieties transforming to original state. Simultaneously, the color changes from colorless to deep red and then, correspondingly, to colorless (Figure 11b inset), indicating the regeneration of the hydrogel fluorescent sensor. The subsequent addition of Fe^3+^ leads to an enhancement in fluorescence emission, indicating that the regenerated hydrogel can still recognize Fe^3+^. As shown in Figure 11b, within 9 Fe^3+^/EDA cycles in aqueous solution, the **P(AAm-co-RBNCH)** still displays a response to Fe^3+^, revealing that the hydrogel chemosensor has excellent reversibility.

## 3. Materials and Methods

### 3.1. Materials

Rhodamine B (99%), ethanediamine (99%), acryloyl chloride (98%), acrylamide (AAm, 99%), N, N′-methylenebisacrylamide (MBA, 99%), N, N, N′, N′-tetramethylethylenediamine (TEMED, 99%), potassium persulfate (99%), ethylenediamine (EDA), Et_3_N, CH_2_Cl_2_, EtOH, DMSO, anhydrous Na_2_SO_4_ and metal salts, such as chloride salts of K^+^, Na^+^, Mg^2+^, Ca^2+^, Zn^2+^, Ni^2+^, Cu^2+^ and Fe^3+^ and nitrate of Mn^2+^, Pb^2+^ and Pr^3+^, were all purchased from Shanghai Aladdin Bio-Chem Technology Co, LTD and used directly as received. All the reagents were of analytical grade, or the highest purity available, and were used without further purification. All solutions (monomer and metal ions) were prepared using CH_3_CN or distilled water.

### 3.2. Instrumental Characterization

NMR spectra were measured and recorded by a Varian instrument (400 MHz) using CDCl_3_ as the solvent and tetramethylsiliane (TMS) as the internal reference. Chemical shifts were expressed in ppm and coupling constants (*J*) in Hz. The Fourier transform infrared radiation (FT-IR) spectra of the hydrogels were recorded with an FT-IR (Perkin Elmer Spectrum 100) instrument using an ATR apparatus with 4 cm^−1^ resolution between 4000 and 650 cm^−1^. The model of mass spectrometer used was a Thermo Scientific Q Exactive, Orbitrap, Waltham, MA, USA. The emission spectra were measured by a Hitach F-4600 fluorescence spectrophotometer, Hitach, Tokyo, Japan.

### 3.3. Synthesis of Monomer **RBNCH**

Synthesis of intermediate **RBNH**: Rhodamine B (2.40 g, 5 mmol) and ethanediamine (1.80 g, 30 mmol) were dissolved in 60 mL EtOH, and refluxed for 12 h. After cooling to ambient temperature, the reaction mixture was added to 60 mL saturated brine solution and extracted with 3 × 20 mL CH_2_Cl_2_. The combined organic phase was dried with anhydrous Na_2_SO_4_ and concentrated by rotary evaporation. The crude product was purified by neutral alumina column, using CH_2_Cl_2_/MeOH (10:1, *v*:*v*) as eluent, to obtain **RBNH** as a pale yellowish solid (1.87 g, 77.8% yield). ^1^H NMR (400 MHz, CDCl_3_) δ 7.89 (dd, *J* = 5.9, 2.8 Hz, 1H), 7.49–7.40 (m, 2H), 7.08 (dd, *J* = 5.8, 2.8 Hz, 1H), 6.43 (d, *J* = 8.8 Hz, 2H), 6.37 (d, *J* = 2.4 Hz, 2H), 6.27 (dd, *J* = 8.9, 2.4 Hz, 2H), 3.33 (q, *J* = 7.1 Hz, 8H), 3.22 (t, *J* = 6.1 Hz, 2H), 2.53 (t, *J* = 6.1 Hz, 2H), 1.16 (t, *J* = 7.0 Hz, 12H).
Synthesis of **RBNCH**: **RBNH** (0.48 g, 1 mmol) and triethylamine (0.15 g, 1.5 mmol) were added to a round bottom flask containing 15 mL dehydrated THF and cooled to 0 °C. Acryloyl chloride (0.11 g, 1.2 mmol) was added to the above solution, dropwise via syringe, and stirred for 12 h at ambient temperature. CH_2_Cl_2_/MeOH (20:1, *v*:*v*) was used as eluent to get the pale yellow solid **RBNCH** (0.43 g, 80.1% yield). ^1^H NMR (400 MHz, CDCl_3_) δ 7.91 (dd, *J* = 5.9, 2.7 Hz, 1H), 7.46 (dd, *J* = 5.4, 3.3 Hz, 2H), 7.18–7.04 (m, 2H), 6.51–6.34 (m, 4H), 6.28 (d, *J* = 7.3 Hz, 2H), 6.19 (dd, *J* = 17.1, 1.4 Hz, 1H), 6.02 (dd, *J* = 17.1, 10.3 Hz, 1H), 5.55 (dd, *J* = 10.3, 1.3 Hz, 1H), 3.34 (q, *J* = 6.8 Hz, 10H), 3.14 (s, 2H), 1.73 (s, 2H), 1.17 (s, 12H). HRMS: [M + H]^+^ (*m*/*z*), 539.2984.

### 3.4. General Procedures for Spectroscopic Analysis of **RBNCH**

The stock solution (5 mM) was obtained by dissolving **RBNCH** in CH_3_CN. The metal ion (K^+^, Na^+^, Mg^2+^, Ca^2+^, Zn^2+^, Ni^2+^, Cu^2+^, Mn^2+^, Pb^2+^ and Pr^3+^) stocks in deionized water were prepared at a concentration of 5 mM. Test solutions were prepared by mixing the stock solution of the **RBNCH** (10 μL) with an appropriate amount of metal ions and then diluting with a mixed solution (CH_3_CN/H_2_O = 9/1, *v*/*v*) to keep the volume of each test solution at 1 mL. The test solutions were shaken for 90 min and then the fluorescence or absorption was measured. Ion recognizing properties were investigated by fluorescent spectrophotometer with the excitation wavelength at 308 nm.

### 3.5. Synthesis and Swelling Properties of **P(AAm-co-RBNCH)** Hydrogel Sensors

According to the synthetic procedure shown in Figure 1, **P(AAm-co-RBNCH)** hydrogels were synthesized via free radical polymerization by different molar ratios of acrylamide and **RBNCH** given in Table 1. Monomer **RBNCH** was dissolved in DMSO, **AAm** was dissolved in deionized water, and both were mixed in a round bottom flask treated with argon gas. Then, cross-linking agent (**MBA**, 0.5 mol% based on total monomer amount), catalyst (**TEMED**, 0.1 mL) and initiator (K_2_S_2_O_8_, 0.1 mol% based on total monomer amount) were added to the above monomer solution. This mixture was stirred until a homogeneous solution formed; the reaction mixture was then transferred to sealed plastic tubes using an injector and kept at 70 °C for 5 h. When the mixture solution lost its flowability, the hydrogel sensor was extracted from the mold, washed with DMSO for 24 h, and later washed with deionized water for 24 h. One piece of each hydrogel was dried in an oven at 40 °C to study the water absorption properties of hydrogel.

The prepared hydrogel’s gel content was determined using a gravimetric method. The untreated hydrogel was dried in an oven at 60 °C to a constant weight (*M_i_*). After washing with DMSO for 24 h and water for 24 h to remove any unreacted monomers and solvent, the sample was dried to a constant weight (*M_d_*). The gel content was calculated using Equation (1):

(1) $$G\%=\frac{M_{d}}{M_{i}}\times 100\%$$

Swelling kinetics of hydrogel sensors were studied by adding a 0.133 g dried hydrogel to deionized water and the increase in mass was measured at certain intervals. The swelling ratio of hydrogel can be calculated according to Equation (2). *M_S_* and *M_D_* in the equation represent the weight of swollen hydrogel and dried hydrogel, respectively.

(2) $$S\%=\frac{M_{S}-M_{D}}{M_{D}}\times 100\%$$

### 3.6. The Application of Hydrogels as Fluorescent Naked-Eye Sensors

The selective recognition of metal ions was proceeded by placing the fully swollen **P(AAm-co-RBNCH)** hydrogels into different metal ion solutions. The detection limits were determined by putting the hydrogel sensors into a range of Fe^3+^ solutions (0.1–200 μM). Furthermore, the reusability of the sensors was investigated by the following process: after the absorption of Fe^3+^, the sensors were soaked in **EDA** solutions for 1 h to form a decolored hydrogel, and then washed by deionized water until neutral pH.

## 4. Conclusions

In summary, polyacrylamide hydrogel sensors with anchored rhodamine dye in the side chain were designed and synthesized via free radical polymerization based on the **RBNCH** monomer. The hydrogels exhibited excellent gelation and swelling ratios in aqueous media, and inherited the ability of the monomer to selectively and sensitively recognize Fe^3+^, which allowed the hydrogels to serve as naked-eye sensors under visible light and UV light. Hydrogel sensors were able to successfully detect 0.1 μM Fe^3+^ in solution, which indicates that the hydrogels could satisfy the demand for monitoring trace Fe^3+^ in the environment. Ultimately, this method provides new insights to profoundly explore smarter sensing systems that synergistically couple visual detection with reusability.

## Figures and Tables

**Table 1:** Synthesis of **P(AAm-co-RBNCH)** hydrogel sensors ^1^.

Hydrogel Code	RBNCH/g	AAm/g	Molar Ratio	H_2_O/mL	DMSO/mL
P-1	0.0807	2.12	0.5/99.5	1.5	6
P-2	0.121	2.11	0.75/99.25	1.5	6
P-3	0.161	2.11	1/99	1.5	6
P-4	0.242	2.10	1.5/98.5	1.5	6
P-5	0.323	2.09	2/98	1.5	6

^1^ Total monomer amount: 0.03 mol, 0.1 mol% K_2_S_2_O_8_, 5 mol% N, N′-Methylenebisacrylamide (**MBA**), 0.1 mL N, N, N′, N′-Tetramethylethylenediamine (**TEMED**).

## Data Availability

Not applicable.

## Supplementary Materials

The following supporting information can be downloaded at: https://www.mdpi.com/article/10.3390/molecules28186572/s1, Figure S1: The ^1^H NMR spectra of **RBNH**; Figure S2: The ^1^H NMR spectra of **RBNCH**; Figure S3: The fluorescence spectra of different water content; Figure S4: The fluorescence spectra of response time; Figure S5: The fluorescence spectra of response time. Fluorescence emission spectra (λ_ex_ = 308 nm) of the sensing system containing 50 μM **RBNCH**, with 100 μM Fe^3+^ for different reaction time; Table S1: Comparison of the proposed method for Fe^3+^ detection with other previously reported fluorescent probes. References [44,45,46,47,48,49] are cited in the Supplementary Materials.
