# Supplementary material for: Rhodamine-Anchored Polyacrylamide Hydrogel for Fluorescent Naked-Eye Sensing of Fe3+
Source: Molecules. 2023 Sep 11;28(18):6572. doi: 10.3390/molecules28186572 (PMC10537437; doi:10.3390/molecules28186572)
Supplement: Supplementary file 1 [file molecules-28-06572-s001.zip › molecules-2559254-supplementary.pdf]

Supporting Information

Rhodamine-anchored polyacrylamide hydrogel for fluorescent naked-eye sensing of Fe^{3+}

Dandan Jiang [1], Minghao Zheng [1], Xiaofan Ma [2], Yingzhen Zhang [1], Shaohua Jiang [2], Juanhua Li [1], Chunmei Zhang [3], Kunming Liu [1,*], Liqing Li [1,*]

[1] Jiangxi Provincial Key Laboratory of Functional Molecular Materials Chemistry, Faculty of Materials Metallurgy and Chemistry, Jiangxi University of Science and Technology, 86 Hongqi Road, Ganzhou 341000, China; 18870773639@163.com (M.-H. Z.); 18720211955@163.com (D.-D. J.); m15779018433@163.com (Y.-Z. Z.); lijuanhua@jxust.edu.cn (J.-H. Li)

[2] Jiangsu Co-Innovation Center of Efficient Processing and Utilization of Forest Resources, International Innovation Center for Forest Chemicals and Materials, College of Materials Science and Engineering, Nanjing Forestry University, Nanjing 210037, China; xiaofan.ma.lab@qq.com (X.-F. M.); Shaohua.jiang@njfu.edu.cn (S.J.)

[3] Institute of Materials Science and Devices, School of Materials Science and Engineering, Suzhou University of Science and Technology, Suzhou 215009, China; cmzhang@usts.edu.cn (C.-M. Z.)

* Correspondence: liukunming@jxust.edu.cn (K.-M. L.); liliqing@jxust.edu.cn (L.-Q. L.)

1 EXPERIMENTAL

1.1 NMR characterization of the product RBNH and RBNCH

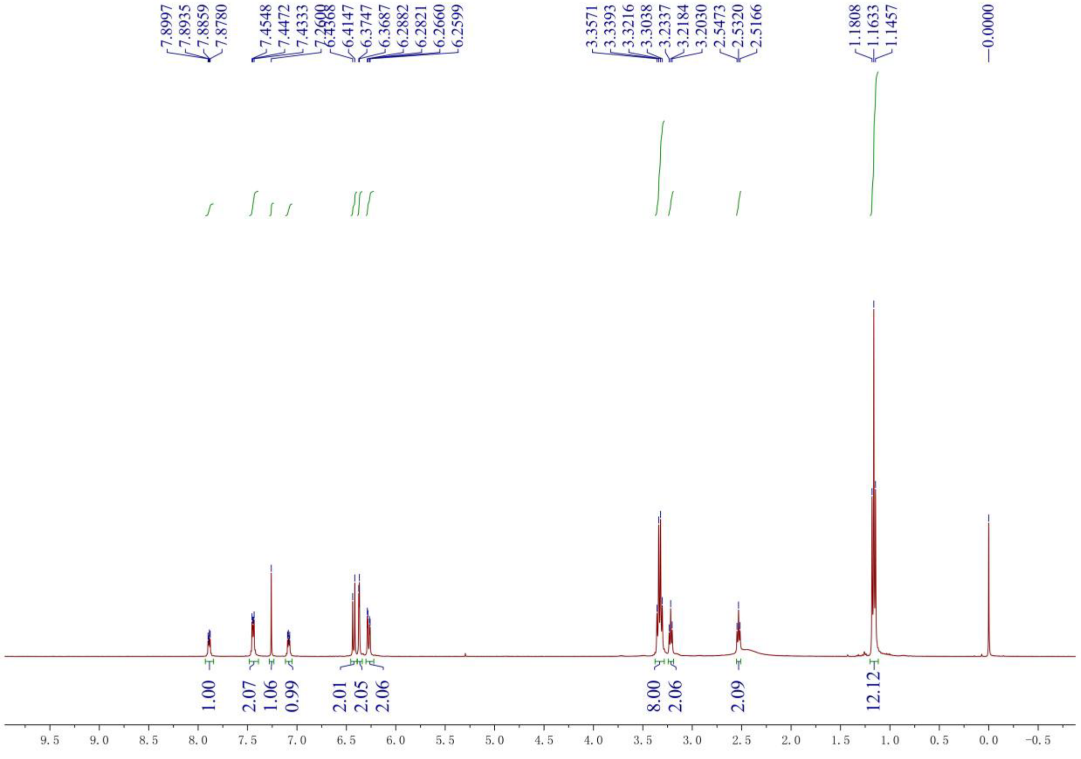

Figure S1. The ^1H NMR spectra of **RBNH**.

The ^1H NMR spectra of **RBNH** was shown as Fig. S1. ^1H NMR (400 MHz, $CDCl_3$) δ 7.89 (dd, *J* = 5.9, 2.8 Hz, 1H), 7.49 – 7.40 (m, 2H), 7.08 (dd, *J* = 5.8, 2.8 Hz, 1H), 6.43 (d, *J* = 8.8

Hz, 2H), 6.37 (d, *J* = 2.4 Hz, 2H), 6.27 (dd, *J* = 8.9, 2.4 Hz, 2H), 3.33 (q, *J* = 7.1 Hz, 8H), 3.22 (t, *J* = 6.1 Hz, 2H), 2.53 (t, *J* = 6.1 Hz, 2H), 1.16 (t, *J* = 7.0 Hz, 12H).

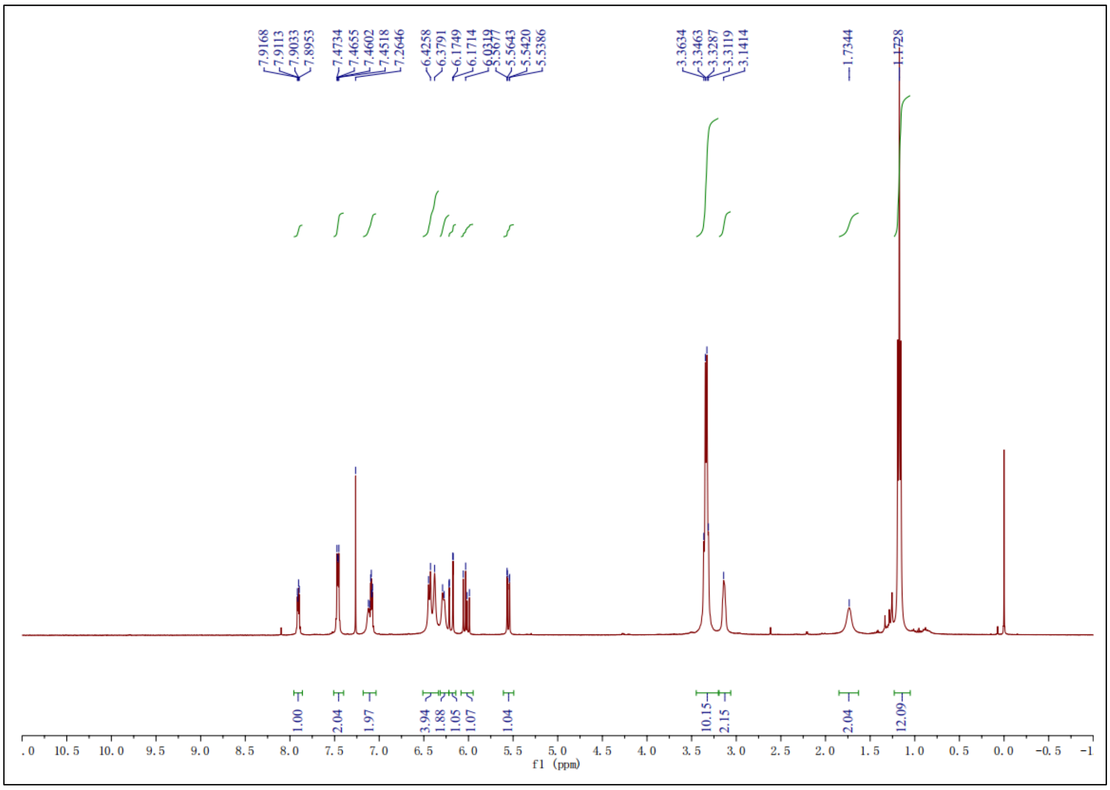

Figure S2. The ^{1}H NMR spectra of probe **RBNCH**.

The ^{1}H NMR spectra of **RBNCH** was shown as Fig. S2. ^{1}H NMR (400 MHz, $CDCl_3$) δ 7.91 (dd, *J* = 5.9, 2.7 Hz, 1H), 7.46 (dd, *J* = 5.4, 3.3 Hz, 2H), 7.18 – 7.04 (m, 2H), 6.51 – 6.34 (m, 4H), 6.28 (d, *J* = 7.3 Hz, 2H), 6.19 (dd, *J* = 17.1, 1.4 Hz, 1H), 6.02 (dd, *J* = 17.1, 10.3 Hz, 1H), 5.55 (dd, *J* = 10.3, 1.3 Hz, 1H), 3.34 (q, *J* = 6.8 Hz, 10H), 3.14 (s, 2H), 1.73 (s, 2H), 1.17 (s, 12H).

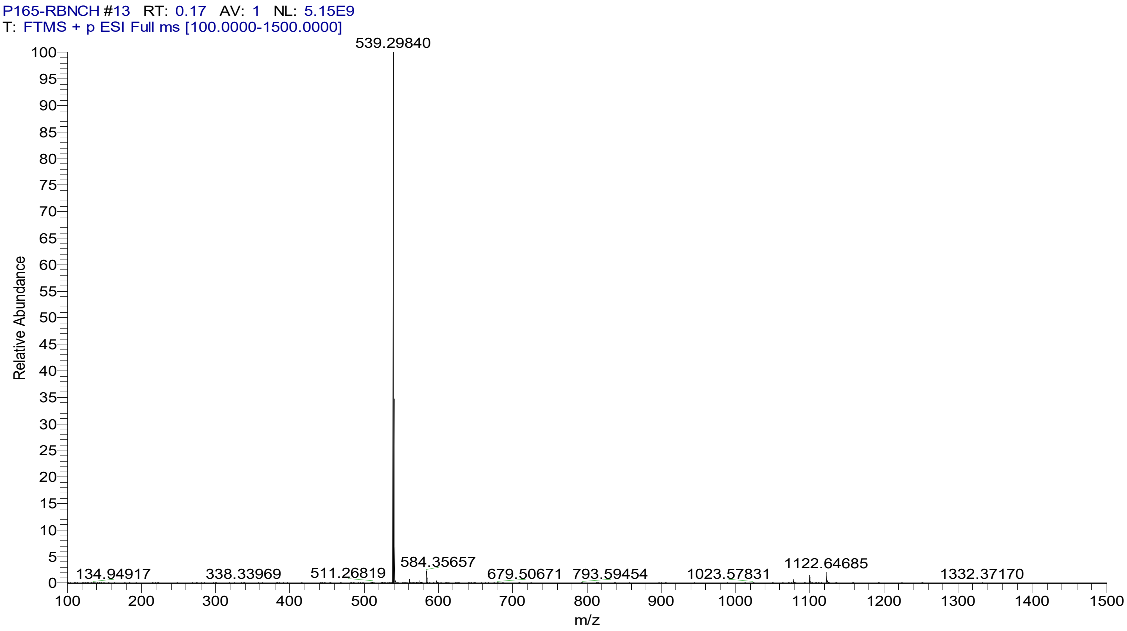

Figure S3. The HRMS spectra of probe **RBNCH**.

1.2 Detection of Fe^{3+} in various percentages of water in CH_3CN

The stock solutions of **RBNCH** (5 mM) were prepared in CH_3CN, $FeCl_3$ (5 mM) were prepared in deionized water. For the detection of Fe^{3+} in various percentages of water in CH_3CN, various amount stock solutions of **RBNCH** and Fe^{3+} were added into dilutes with H_2O/CH_3CN ratio range from (9:1) to (1:9), diluting to the testing solution consist of **RBNCH** (100 μM) and Fe^{3+} (100 μM). Then the mixture was incubated at room temperature for 1.5 h. Emission spectra were measured in the range of 385 nm to 700 nm with an excitation wavelength at 308 nm, and the slit width is 10 nm/10 nm.

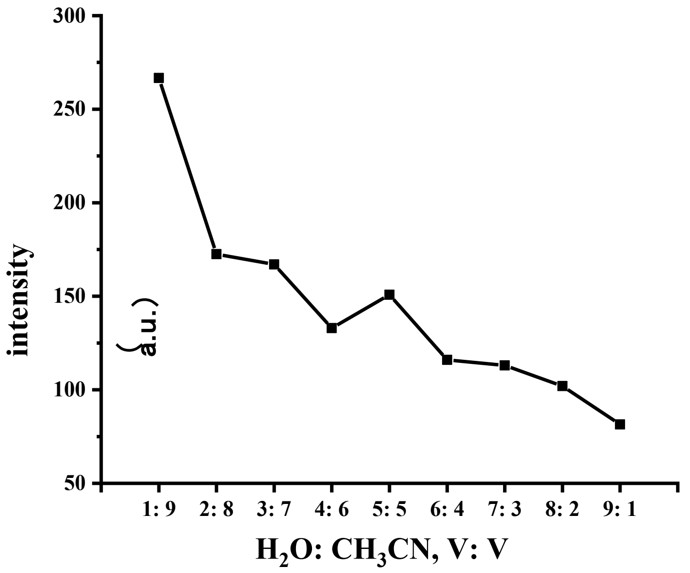

Figure S4. The fluorescence spectra of different water content. Fluorescence emission spectra (λex = 308 nm) of the sensing system containing 50 μM RBNCH, with 100 μM Fe^{3+} in different ratios of H_2O/CH_3CN.

1.3 Detection of Fe^{3+} in different fluorescence response time.

RBNCH (50 μM), $FeCl_3$ (100 μM) were added into various percentages of water in CH_3CN (water content 10%), and then the mixture was incubated at room temperature to test the fluorescence response at different time. Emission spectra were measured in the range of 385 nm to 700 nm with an excitation wavelength at 308 nm, and the slit width is 10 nm/10 nm.

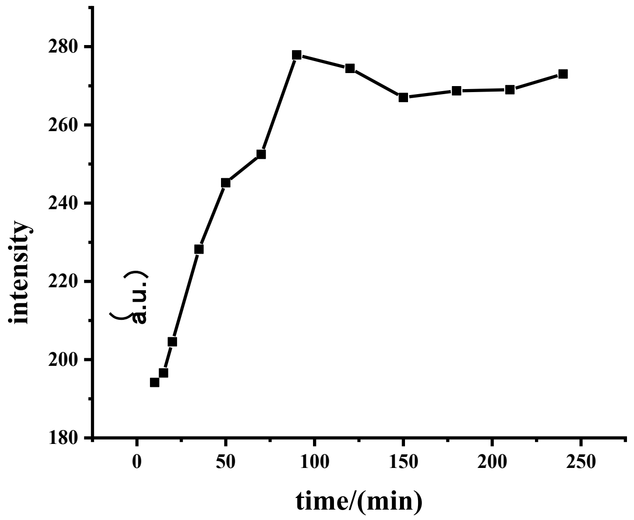

Figure S5. The fluorescence spectra of response time. Fluorescence emission spectra (λex = 308 nm) of the sensing system containing 50 μM RBNCH, with 100 μM Fe^{3+} for different reaction time.

2. COMPARISON OF THE FLUORESCENT PROBE WITH OTHER REPORTED PORBES.

The sensitivity data of several fluorescent probes for the detection of Fe^{3+} were compared with **RBNCH** as shown in table S1. It can be seen that **RBNCH** possesses the wider linear scope and higher sensitivity compared with the reported probes.

Table S1. Comparison of the proposed method for Fe^{3+} detection with other previously reported fluorescent probes.

Entry	Probe	Ex/Em (nm)	Linear range (μM)	LOD (nM)	Ref.
1	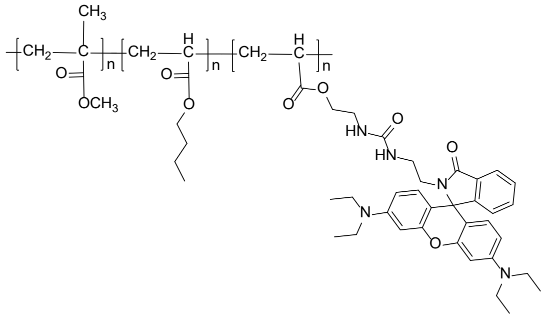	500/579	0-25	122	[1]

2	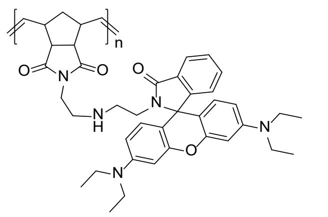	520/580	0-200	19	[2]
3	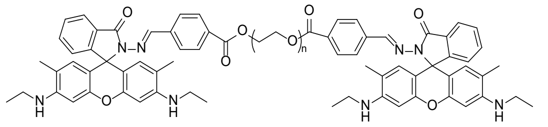	394/555	40-200	1×10^3	[3]
4	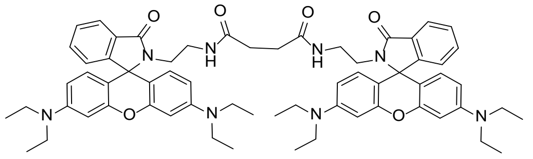	505/585	0.3-14	12.4	[4]
5	An optical fiber sensor based on fluorescent carbon dots	370/475	100-1000	3×10^5	[5]
6	Highly fluorescent carbon polymer dots	350/430	0.2-10	100	[6]
7	Fluorochromic hydrogels	550/665	1-40	150	[7]
8	Carbon quantum dots-based fluorescent hydrogel hybrid	370/450	0-150	240	[8]
**9**	**RBNCH (this work)**	**308/586**	**100-200**	**27**	**Thiswork**

References

1. Liu, X.; Chen, A.; Wu, Y.; Kan, C.; Xu, J. Fabrication of fluorescent polymer latexes based on Rhodamine B derivatives and their reusable films for Fe^{3+} detection. *Dyes Pigments.* **2020**, *182*, 108633.
2. Samanta, T.; Das, N.; Singha, J.; Shunmugam, R. Unusual Red-OrangeEmission from Rhodamine-Derived Polynorbornene for Selective Binding to Fe^{3+} Ions in an Aqueous Environment. *Anal. Methods* **2020**, *12*, 4159–4165.
3. Qiu, X.-Y.; Huang, J.; Wang, N.; Zhao, K.-J.; Cui, J.-W.; Hao, J.-C. Facile synthesis of water-soluble rhodamine-based polymeric chemosensors via schiff base reaction for Fe^{3+} detection and living cell imaging. *Front. Chem.* **2022**, *10*, 845627.
4. Luo, A.; Wang, H.; Wang, Y.; Huang, Q.; Zhang, Q. A novel colorimetric and turn-on fluorescent chemosensor for iron(III) ion detection and its application to cellular imaging. *Spectrochim. Acta A: Mol. Biomol. Spectrosc.* **2016**, *168*, 37–44.
5. Lin, H.; Huang, J.; Ding, L.; A recyclable optical fiber sensor based on fluorescent carbon dots for the determination of ferric ion concentrations. *J. Lightwave Technol.* **2019**, *37*, 4815-4822.
6. Xia, J.; Zhuang, Y.-T.; Yu, Y.-L.; Wang, J.-H. Highly fluorescent carbon polymer dots prepared at room temperature, and their application as a fluorescent probe for determination and intracellular imaging of ferric ion. *Microchim. Acta.* **2017**, *184*, 1109-1116.
7. Wang, B.-B.; Liu, L.-J.; Liao, L.-Q. Light and ferric ion responsive fluorochromic hydrogels with high strength and self-healing ability. *Polym. Chem.* **2019**, *10*, 6481-6488.
8. Wu, S.-W.; Zhou, C.; Ma, C.-M.; Yin, Y.-Z.; Sun, C.-Y. Carbon quantum dots-based fluorescent hydrogel hybrid platform for sensitive detection of iron ions. *J. Chem.* **2022**, *2022*, 3737646.
